# Supplementary material for: Genetic variants in the leptin-melanocortin pathway and their joint effects with physical activity and sleep duration on risk of childhood obesity
Source: PLoS One. 2026 May 15;21(5):e0348694. doi: 10.1371/journal.pone.0348694 (PMC13178977; doi:10.1371/journal.pone.0348694)
Supplement: S1 Table — (DOCX) [file pone.0348694.s002.docx]

**S1 Table.** Characteristics of the 12 genetic variants in leptin-melanocortin pathway

| Genes | SNPs | Chromosome location | Functional region in gene | Minor  allele | MAF in CHS/CHB^a^ | MAF in controls | Call rate (%) | *P* for HWE |
| --- | --- | --- | --- | --- | --- | --- | --- | --- |
| *LEP* | rs1349419 | 7:127877213 | 5’UTR | A | 0.286/0.238 | 0.277 | 99.96 | 0.465 |
| *LEP* | rs2167270 | 7:127881349 | 5’UTR | A | 0.219/0.214 | 0.218 | 99.92 | 0.949 |
| *LEPR* | rs11208659 | 1:65979280 | intronic | C | 0.038/0.073 | 0.061 | 100.00 | 0.930 |
| *LEPR* | rs1137100 | 1:66036441 | exon_4 | A | 0.157/0.194 | 0.160 | 100.00 | 0.978 |
| *LEPR* | rs1137101 | 1:66058513 | exon_6 | A | 0.124/0.131 | 0.105 | 99.96 | 0.970 |
| *POMC* | rs6713532 | 2:25384833 | intronic | T | 0.376/0.340 | 0.408 | 100.00 | 0.225 |
| *NPY* | rs16141 | 7:24324759 | intronic | T | 0.343/0.311 | 0.362 | 99.96 | 0.288 |
| *MC3R* | rs6127698 | 20:54823416 | 5’UTR | G | 0.357/0.325 | 0.334 | 99.92 | 0.212 |
| *MC3R* | rs3746619 | 20:54823805 | 5’UTR | A | 0.238/0.223 | 0.225 | 99.92 | 0.039^b^ |
| *MC4R* | rs17782313 | 18:57851097 | 3’UTR | C | 0.157/0.184 | 0.163 | 100.00 | 0.098 |
| *MC4R* | rs12970134 | 18:57884750 | 3’UTR | A | 0.171/0.175 | 0.160 | 99.83 | 0.181 |
| *MC4R* | rs8087522 | 18:58040478 | 5’UTR | A | 0.143/0.097 | 0.128 | 99.96 | 0.722 |

CHB, Han Chinese in Beijing, China; CHS, Han Chinese South, China; HWE, Hardy-Weinberg Equilibrium; MAF, minor allele frequency; SNPs, single‐nucleotide polymorphisms. ^a^ MAF for CHS and CHB in the 1000 Genomes Project.

^b^ The *P* value for Hardy-Weinberg equilibrium corrected by permutation test was 0.046.
