# Supplementary material for: Genetic variants in the leptin-melanocortin pathway and their joint effects with physical activity and sleep duration on risk of childhood obesity
Source: PLoS One. 2026 May 15;21(5):e0348694. doi: 10.1371/journal.pone.0348694 (PMC13178977; doi:10.1371/journal.pone.0348694)
Supplement: S2 Table — (DOCX) [file pone.0348694.s003.docx]

**S2 Table.** Functional annotations of the 12 genetic variants in leptin-melanocortin pathway

| SNPs | SNPinfo | Polyphen | Regulome  -DB score | HaploReg v4.2 | | | | | |
| --- | --- | --- | --- | --- | --- | --- | --- | --- | --- |
|  |  |  |  | Promoter histone marks | Enhancer histone marks | Proteins bound | Motifs changed | eQtL hits | Correlated gene |
| rs1349419 | - | - | 1f | SKIN, LNG | 12 tissues (FAT) | 12 bound proteins | Elf3, Ets, Spdef | 5 | LEP |
| rs2167270 | - | - | 1b | FAT, BLD | 8 tissues (FAT) | CEBPB | AP-4, BCL, Sin3Ak-20 | 1 | LEP |
| rs11208659 | - | - | 4 | - | 11 tissues (FAT) | CFOS,  P300 | NF-kappaB | 3 | LEPR |
| rs1137100 | - | Missense,  possibly damaging | 7 | - | - | - | - | 5 | LEPR |
| rs1137101 | - | Missense,  possibly damaging | 5 | - | - | - | - | 2 | LEPR |
| rs6713532 | - | - | 1b | 7 tissues (FAT) | 7 tissues (FAT) | - | TATA_disc7 | 2 | POMC |
| rs16141 | - | - | 1f | 8 tissues (FAT) | 15 tissues (FAT) | CTCF, RAD21 | 4 altered motifs | - | - |
| rs6127698 | Affecting  TFBS | - | 1f | BRST | IPSC, SKIN, PLCNT | - | DMRT5, Spz1 | 1 | C20orf108 |
| rs3746619 | - | - | 4 | BRST | - | - | FAC1 | 1 | CSTF1 |
| rs17782313 | - | - | 7 | - | - | - | Pou5f1 | 6 | CTNNA1 |
| rs12970134 | - | - | 1f | SKIN | - | - | 12 altered motifs | 6 | CTNNA1 |
| rs8087522 | - | - | 1f | 7 tissues (FAT) | 7 tissues (FAT) | - | 6 altered motifs | - | - |

eQtL, expression quantitative trait loci; LEP, leptin; LEPR, leptin receptor; POMC, pro-opiomelanocortin; SNP, single‐nucleotide polymorphism; TFBS, transcription factor binding site.
