# Supplementary material for: Genetic variants in the leptin-melanocortin pathway and their joint effects with physical activity and sleep duration on risk of childhood obesity
Source: PLoS One. 2026 May 15;21(5):e0348694. doi: 10.1371/journal.pone.0348694 (PMC13178977; doi:10.1371/journal.pone.0348694)
Supplement: S3 Table — (DOCX) [file pone.0348694.s004.docx]

**S3 Table.** Genotypic distribution of 12 genetic variants in leptin-melanocortin pathway in cases and controls

| Genotypes | Cases (%)  N=1123 | Controls (%) N=1231 | *χ^2^* | *P* |
| --- | --- | --- | --- | --- |
| *LEP* rs1349419^a^ |  |  | 1.31 | 0.520 |
| GG | 608 (54.14) | 637 (51.79) |  |  |
| AG | 437 (38.91) | 504 (40.98) |  |  |
| AA | 78 (6.95) | 89 (7.24) |  |  |
| *LEP* rs2167270^a^ |  |  | 0.16 | 0.924 |
| GG | 694 (61.80) | 753 (61.27) |  |  |
| AG | 373 (33.21) | 417 (33.93) |  |  |
| AA | 56 (4.99) | 59 (4.80) |  |  |
| *LEPR* rs11208659 |  |  | 0.08 | 0.985 |
| TT | 991 (88.25) | 1084 (88.06) |  |  |
| CT | 129 (11.49) | 143 (11.62) |  |  |
| CC | 3 (0.27) | 4 (0.32) |  |  |
| *LEPR* rs1137100 |  |  | 1.01 | 0.605 |
| GG | 778 (69.28) | 869 (70.59) |  |  |
| AG | 310 (27.60) | 331 (26.89) |  |  |
| AA | 35 (3.12) | 31 (2.52) |  |  |
| *LEPR* rs1137101^a^ |  |  | 1.57 | 0.455 |
| GG | 881 (78.45) | 985 (80.08) |  |  |
| AG | 225 (20.04) | 232 (18.86) |  |  |
| AA | 17 (1.51) | 13 (1.06) |  |  |
| *POMC* rs6713532 |  |  | 3.45 | 0.178 |
| CC | 364 (32.41) | 435 (35.34) |  |  |
| CT | 579 (51.56) | 588 (47.77) |  |  |
| TT | 180 (16.03) | 208 (16.90) |  |  |
| *NPY* rs16141^a^ |  |  | 1.81 | 0.405 |
| GG | 467 (41.59) | 492 (40.00) |  |  |
| GT | 505 (44.97) | 586 (47.64) |  |  |
| TT | 151 (13.45) | 152 (12.36) |  |  |
| *MC3R* rs6127698^a^ |  |  | 1.00 | 0.606 |
| TT | 508 (45.24) | 535 (43.53) |  |  |
| GT | 495 (44.08) | 567 (46.14) |  |  |
| GG | 120 (10.69) | 127 (10.33) |  |  |
| *MC3R* rs3746619^a^ |  |  | 1.79 | 0.409 |
| CC | 665 (59.22) | 726 (59.07) |  |  |
| AC | 401 (35.71) | 454 (36.94) |  |  |
| AA | 57 (5.08) | 49 (3.99) |  |  |
| *MC4R* rs17782313 |  |  | 14.77 | <0.001 |
| TT | 711 (63.31) | 871 (70.76) |  |  |
| CT | 366 (32.59) | 319 (25.91) |  |  |
| CC | 46 (4.10) | 41 (3.33) |  |  |
| *MC4R* rs12970134^a^ |  |  | 8.25 | 0.016 |
| GG | 739 (65.86) | 874 (71.17) |  |  |
| AG | 335 (29.86) | 316 (25.73) |  |  |
| AA | 48 (4.28) | 38 (3.09) |  |  |
| *MC4R* rs8087522^a^ |  |  | 1.03 | 0.598 |
| GG | 869 (77.38) | 937 (76.18) |  |  |
| AG | 239 (21.28) | 271 (22.03) |  |  |
| AA | 15 (1.34) | 22 (1.79) |  |  |

^a^ Variables with missing data.
