# Supplementary material for: Genetic variants in the leptin-melanocortin pathway and their joint effects with physical activity and sleep duration on risk of childhood obesity
Source: PLoS One. 2026 May 15;21(5):e0348694. doi: 10.1371/journal.pone.0348694 (PMC13178977; doi:10.1371/journal.pone.0348694)
Supplement: S4 Table — (DOCX) [file pone.0348694.s005.docx]

**S4 Table.** Association between 10 genetic variants in leptin-melanocortin pathway and obesity risk in Chinese children and adolescents

| Genotypes | *OR* (95% *CI*) | *P* | *OR* (95% *CI*)^b^ | *P*^b^ |
| --- | --- | --- | --- | --- |
| *LEP* rs1349419^a^ |  |  |  |  |
| GG | 1.00 |  | 1.00 |  |
| AG | 0.91 (0.77-1.08) | 0.267 | 0.91 (0.76-1.07) | 0.250 |
| AA | 0.92 (0.66-1.27) | 0.605 | 0.91 (0.66-1.26) | 0.574 |
| Dominant model | 0.91 (0.77-1.07) | 0.254 | 0.91 (0.77-1.07) | 0.234 |
| Recessive model | 0.96 (0.70-1.31) | 0.785 | 0.95 (0.69-1.31) | 0.757 |
| Additive model | 0.94 (0.82-1.06) | 0.306 | 0.93 (0.82-1.06) | 0.280 |
| *LEP* rs2167270^a^ |  |  |  |  |
| GG | 1.00 |  | 1.00 |  |
| AG | 0.97 (0.82-1.16) | 0.736 | 0.97 (0.81-1.15) | 0.710 |
| AA | 1.03 (0.70-1.51) | 0.879 | 1.02 (0.70-1.50) | 0.901 |
| Dominant model | 0.98 (0.83-1.16) | 0.792 | 0.98 (0.83-1.15) | 0.762 |
| Recessive model | 1.04 (0.72-1.51) | 0.834 | 1.04 (0.71-1.51) | 0.852 |
| Additive model | 0.99 (0.86-1.14) | 0.887 | 0.99 (0.86-1.13) | 0.855 |
| *LEPR* rs11208659 |  |  |  |  |
| TT | 1.00 |  | 1.00 |  |
| CT | 0.99 (0.77-1.27) | 0.918 | 0.99 (0.77-1.27) | 0.911 |
| CC | 0.82 (0.18-3.68) | 0.797 | 0.79 (0.18-3.53) | 0.753 |
| Dominant model | 0.98 (0.77-1.26) | 0.888 | 0.98 (0.76-1.26) | 0.875 |
| Recessive model | 0.82 (0.18-3.68) | 0.799 | 0.79 (0.18-3.53) | 0.755 |
| Additive model | 0.98 (0.77-1.25) | 0.860 | 0.98 (0.77-1.24) | 0.840 |
| *LEPR* rs1137100 |  |  |  |  |
| GG | 1.00 |  | 1.00 |  |
| AG | 1.05 (0.87-1.26) | 0.629 | 1.05 (0.87-1.26) | 0.601 |
| AA | 1.26 (0.77-2.07) | 0.356 | 1.26 (0.77-2.06) | 0.366 |
| Dominant model | 1.07 (0.89-1.27) | 0.487 | 1.07 (0.90-1.27) | 0.468 |
| Recessive model | 1.25 (0.76-2.03) | 0.380 | 1.24 (0.76-2.02) | 0.393 |
| Additive model | 1.07 (0.92-1.25) | 0.378 | 1.07 (0.92-1.25) | 0.367 |
| *LEPR* rs1137101^a^ |  |  |  |  |
| GG | 1.00 |  | 1.00 |  |
| AG | 1.08 (0.88-1.33) | 0.438 | 1.09 (0.89-1.34) | 0.409 |
| AA | 1.46 (0.71-3.02) | 0.308 | 1.46 (0.71-3.03) | 0.306 |
| Dominant model | 1.10 (0.91-1.35) | 0.329 | 1.11 (0.91-1.36) | 0.306 |
| Recessive model | 1.44 (0.70-2.97) | 0.328 | 1.44 (0.70-2.98) | 0.327 |
| Additive model | 1.11 (0.93-1.33) | 0.254 | 1.12 (0.93-1.34) | 0.237 |
| *POMC* rs6713532 |  |  |  |  |
| CC | 1.00 |  | 1.00 |  |
| CT | 1.18 (0.98-1.41) | 0.077 | 1.18 (0.98-1.41) | 0.076 |
| TT | 1.03 (0.81-1.32) | 0.788 | 1.04 (0.81-1.32) | 0.778 |
| Dominant model | 1.14 (0.96-1.35) | 0.135 | 1.14 (0.96-1.35) | 0.132 |
| Recessive model | 0.94 (0.76-1.17) | 0.571 | 0.94 (0.76-1.17) | 0.578 |
| Additive model | 1.04 (0.93-1.18) | 0.469 | 1.05 (0.93-1.18) | 0.463 |
| *NPY* rs16141^a^ |  |  |  |  |
| GG | 1.00 |  | 1.00 |  |
| GT | 0.91 (0.76-1.08) | 0.276 | 0.90 (0.76-1.07) | 0.246 |
| TT | 1.05 (0.81-1.36) | 0.729 | 1.04 (0.80-1.34) | 0.783 |
| Dominant model | 0.94 (0.79-1.10) | 0.434 | 0.93 (0.79-1.10) | 0.387 |
| Recessive model | 1.10 (0.87-1.40) | 0.431 | 1.10 (0.86-1.40) | 0.458 |
| Additive model | 0.99 (0.88-1.12) | 0.859 | 0.98 (0.87-1.11) | 0.795 |
| *MC3R* rs6127698^a^ |  |  |  |  |
| TT | 1.00 |  | 1.00 |  |
| GT | 0.92 (0.78-1.09) | 0.336 | 0.92 (0.77-1.09) | 0.326 |
| GG | 1.00 (0.75-1.31) | 0.672 | 0.99 (0.75-1.31) | 0.938 |
| Dominant model | 0.93 (0.79-1.10) | 0.406 | 0.93 (0.79-1.10) | 0.390 |
| Recessive model | 1.04 (0.80-1.35) | 0.781 | 1.03 (0.79-1.35) | 0.811 |
| Additive model | 0.97 (0.86-1.10) | 0.619 | 0.97 (0.86-1.09) | 0.591 |
| *MC3R* rs3746619^a^ |  |  |  |  |
| CC | 1.00 |  | 1.00 |  |
| AC | 0.96 (0.81-1.14) | 0.676 | 0.96 (0.81-1.14) | 0.646 |
| AA | 1.27 (0.86-1.89) | 0.237 | 1.27 (0.85-1.89) | 0.239 |
| Dominant model | 0.99 (0.84-1.17) | 0.943 | 0.99 (0.84-1.17) | 0.912 |
| Recessive model | 1.29 (0.87-1.90) | 0.204 | 1.29 (0.87-1.90) | 0.204 |
| Additive model | 1.03 (0.90-1.18) | 0.694 | 1.03 (0.89-1.18) | 0.718 |
| *MC4R* rs8087522^a^ |  |  |  |  |
| GG | 1.00 |  | 1.00 |  |
| AG | 0.95 (0.78-1.16) | 0.617 | 0.95 (0.78-1.16) | 0.641 |
| AA | 0.74 (0.38-1.43) | 0.363 | 0.72 (0.37-1.41) | 0.341 |
| Dominant model | 0.94 (0.77-1.13) | 0.491 | 0.94 (0.77-1.14) | 0.505 |
| Recessive model | 0.74 (0.38-1.44) | 0.379 | 0.73 (0.38-1.42) | 0.355 |
| Additive model | 0.93 (0.78-1.10) | 0.391 | 0.93 (0.78-1.10) | 0.395 |

*CI*, confidence interval; *OR*, odds ratio.

^a^ Variables with missing data.

^b^ Multivariate logistic regression models were adjusted for grade, sex, maternal and paternal education levels, and household incomes.
