# Supplementary material for: Genetic variants in the leptin-melanocortin pathway and their joint effects with physical activity and sleep duration on risk of childhood obesity
Source: PLoS One. 2026 May 15;21(5):e0348694. doi: 10.1371/journal.pone.0348694 (PMC13178977; doi:10.1371/journal.pone.0348694)
Supplement: S5 Table — (DOCX) [file pone.0348694.s006.docx]

**S5 Table.** Obesity risk estimates of the terminal nodes in CART among Chinese children and adolescents

| Node | Factors contained in the terminal nodes | Proportion  of cases (%) | *OR* (95% *CI*)^a^ | *P*^a^ |
| --- | --- | --- | --- | --- |
| 12 | Rs17782313 TT, rs1349419 GG, rs8087522 GG, rs1137101 AG/AA | 42.1 | 1 |  |
| 8 | Rs17782313 TT, rs1349419 GG, rs8087522 AG/AA | 42.8 | 1.02 (0.66-1.58) | 0.928 |
| 4 | Rs17782313 TT, rs1349419 AG/AA | 43.5 | 1.05 (0.74-1.50) | 0.790 |
| 10 | Rs17782313 CT/CC, rs6713532 CC, rs1137101 GG | 44.2 | 1.07 (0.70-1.66) | 0.745 |
| 14 | Rs17782313 TT, rs1349419 GG, rs8087522 GG, rs1137101 GG, rs16141 GT/TT | 46.6 | 1.19 (0.80-1.76) | 0.401 |
| 15 | Rs17782313 TT, rs1349419 GG, rs8087522 GG, rs1137101 GG, rs16141 GG | 51.9 | 1.49 (0.98-2.27) | 0.064 |
| 7 | Rs17782313 CT/CC, rs6713532 CT/TT | 56.4 | 1.77 (1.23-2.55) | 0.002 |
| 11 | Rs17782313 CT/CC, rs6713532 CC, rs1137101 AG/AA | 56.9 | 1.82 (0.96-3.45) | 0.069 |

CART, classification and regression tree; *CI*, confidence interval; *OR*, odds ratio.

^a^ Multivariate logistic regression models were adjusted for age, sex, maternal and paternal education levels, and household incomes.
