# Supplementary material for: Genetic variants in the leptin-melanocortin pathway and their joint effects with physical activity and sleep duration on risk of childhood obesity
Source: PLoS One. 2026 May 15;21(5):e0348694. doi: 10.1371/journal.pone.0348694 (PMC13178977; doi:10.1371/journal.pone.0348694)
Supplement: S6 Table — (DOCX) [file pone.0348694.s007.docx]

**S6 Table.** Association between GRS (high vs. low/medium) and obesity risk in Chinese children and adolescents by sex and age group

| Stratified variables | *OR* (95% *CI*) | *OR* (95% *CI*)^a^ | *P*_heterogeneity_^b^ |
| --- | --- | --- | --- |
| Unweighted GRS |  |  |  |
| Sex |  |  | 0.760 |
| Boys | 1.37 (1.10-1.71) | 1.38 (1.11-1.72) |  |
| Girls | 1.47 (1.07-2.03) | 1.47 (1.07-2.03) |  |
| Age groups |  |  | 0.153 |
| 7-8 years | 1.83 (1.19-2.81) | 1.88 (1.21-2.91) |  |
| 11-18 years | 1.32 (1.08-1.61) | 1.32 (1.08-1.62) |  |
| Weighted GRS |  |  |  |
| Sex |  |  | 0.812 |
| Boys | 1.34 (1.08-1.67) | 1.35 (1.08-1.67) |  |
| Girls | 1.27 (0.93-1.74) | 1.28 (0.94-1.76) |  |
| Age groups |  |  | 0.301 |
| 7-8 years | 1.60 (1.06-2.42) | 1.62 (1.06-2.46) |  |
| 11-18 years | 1.26 (1.03-1.54) | 1.26 (1.04-1.54) |  |

*CI*, confidence interval; GRS, genetic risk score; *OR*, odds ratio.

^a^ Multivariate logistic regression models were adjusted for grade, sex, maternal and paternal education levels, and household incomes.

^b^ *P* values for heterogeneities in the *β* coefficients of GRS on obesity risk between subgroups using the *Z*-test.
