# Supplementary material for: Genetic variants in the leptin-melanocortin pathway and their joint effects with physical activity and sleep duration on risk of childhood obesity
Source: PLoS One. 2026 May 15;21(5):e0348694. doi: 10.1371/journal.pone.0348694 (PMC13178977; doi:10.1371/journal.pone.0348694)
Supplement: S1 Text — (DOCX) [file pone.0348694.s008.docx]

**S1 Text. Statistical analytical code.**

**1. Multivariate logistic regression models were used to analyze the association between genetic variants and obesity risk, adjusting for age, sex, maternal and paternal education levels, and household income. The analytical code for SAS 9.4 is as follows:**

1. **Codominant model**

%let data=mydata_codominant; %let x=rs1349419; %let y=obesity;

*The following 11 SNPs were sequentially entered as x: rs2167270, rs11208659, rs1137100, rs1137101, rs6713532, rs16141, rs6127698, rs3746619, rs17782313, rs12970134, and rs8087522;

proc logistic desc data=&data;

class &x /param=ref Ref=first;

model &y(ref='0')=&x;

title "Crude model"; run;

proc logistic desc data=&data;

class &x /param=ref Ref=first;

model &y(ref='0')=&x age sex maternal paternal incomes;

title "Adjusted model"; run;

1. **Dominant model**

%let data=mydata_dominant; %let x=rs1349419; %let y=obesity;

*The following 11 SNPs were sequentially entered as x: rs2167270, rs11208659, rs1137100, rs1137101, rs6713532, rs16141, rs6127698, rs3746619, rs17782313, rs12970134, and rs8087522;

**proc** **logistic** desc data=&data;

model &y(ref='0')=&x;

title "Crude model"; **run**;

**proc** **logistic** desc data=&data;

model &y(ref='0')=&x age sex maternal paternal incomes;

title "Adjusted model"; **run**;

1. **Recessive model**

%let data=mydata_recessive; %let x=rs1349419; %let y=obesity;

*The following 11 SNPs were sequentially entered as x: rs2167270, rs11208659, rs1137100, rs1137101, rs6713532, rs16141, rs6127698, rs3746619, rs17782313, rs12970134, and rs8087522;

**proc** **logistic** desc data=&data;

model &y(ref='0')=&x;

title "Crude model"; **run**;

**proc** **logistic** desc data=&data;

model &y(ref='0')=&x age sex maternal paternal incomes;

title "Adjusted model"; **run**;

1. **Additive model**

%let data=mydata_additive; %let x=rs1349419; %let y=obesity;

*The following 11 SNPs were sequentially entered as x: rs2167270, rs11208659, rs1137100, rs1137101, rs6713532, rs16141, rs6127698, rs3746619, rs17782313, rs12970134, and rs8087522;

**proc** **logistic** desc data=&data;

model &y(ref='0')=&x;

title "Crude model"; **run**;

**proc** **logistic** desc data=&data;

model &y(ref='0')=&x age sex maternal paternal incomes;

title "Adjusted model"; **run**;

**2. A 1000 times permutation test was conducted for correction of multiple comparisons in the genetic association analyses. The analytical code for R v4.2.3 is as follows:**

**（1）Codominant model，rs17782313：**

library('glmperm')

mydata$rs17782313_1 <- relevel(mydata$rs17782313, ref="TT")

prr.test(formula =obesity~rs17782313_1+ age + sex + maternal + paternal + incomes, var="rs17782313_1CT", family=binomial(link='logit'), data=mydata, nrep = 1000, seed=12345)

prr.test(formula =obesity~rs17782313_1+ age + sex + maternal + paternal + incomes, var="rs17782313_1CC", family=binomial(link='logit'), data=mydata, nrep = 1000, seed=12345)

**（2）Codominant model，rs12970134：**

mydata$rs12970134_1 <- relevel(mydata$rs12970134, ref="GG")

prr.test(formula =obesity~ rs12970134_1+ age + sex + maternal + paternal + incomes, var="rs12970134_1AG", family=binomial(link='logit'), data=mydata, nrep = 1000, seed=12345)

prr.test(formula =obesity~ rs12970134_1+ age + sex + maternal + paternal + incomes, var="rs12970134_1AA", family=binomial(link='logit'), data=mydata, nrep = 1000, seed=12345)

**（3）Dominant model，rs17782313：**

mydata$rs17782313_2 <- ifelse(mydata$rs17782313 == "TT", 0, 1)

mydata$rs17782313_2 <- factor(mydata$rs17782313_2)

prr.test(formula =obesity~rs17782313_2+age + sex + maternal + paternal + incomes, var="rs17782313_21", family=binomial(link='logit'), data=mydata, nrep = 1000, seed=12345)

**（4）Dominant model，rs12970134：**

mydata$rs12970134_2 <- ifelse(mydata$rs12970134 == "GG", 0, 1)

mydata$rs12970134_2 <- factor(mydata$rs12970134_2)

prr.test(formula =obesity~rs12970134_2+age + sex + maternal + paternal + incomes, var="rs12970134_21", family=binomial(link='logit'), data=mydata, nrep = 1000, seed=12345)

**（5）Recessive model，rs17782313：**

mydata$rs17782313_3 <- ifelse(mydata$rs17782313 == "CC", 1, 0)

mydata$rs17782313_3 <- factor(mydata$rs17782313_3)

prr.test(formula =obesity~rs17782313_3+age + sex + maternal + paternal + incomes, var="rs17782313_31", family=binomial(link='logit'), data=mydata, nrep = 1000, seed=12345)

**（6）Recessive model，rs12970134：**

mydata$rs12970134_3 <- ifelse(mydata$rs12970134 == "AA", 1, 0)

mydata$rs12970134_3 <- factor(mydata$rs12970134_3)

prr.test(formula =obesity~rs12970134_3+age + sex + maternal + paternal + incomes, var="rs12970134_31", family=binomial(link='logit'), data=mydata, nrep = 1000, seed=12345)

**（7）Additive model，rs17782313：**

mydata$rs17782313_4 <- ifelse(mydata$rs17782313 == "TT", 1,

ifelse(mydata$rs17782313 == "CT", 2, 3))

prr.test(formula =obesity~rs17782313_4+age + sex + maternal + paternal + incomes, var="rs17782313_4", family=binomial(link='logit'), data=mydata, nrep = 1000, seed=12345))

**（8）Additive model，rs12970134：**

mydata$rs12970134_4 <- ifelse(mydata$rs12970134 == "GG", 1,

ifelse(mydata$rs12970134 == "AG", 2, 3))

prr.test(formula =obesity~rs12970134_4+age + sex + maternal + paternal + incomes, var="rs12970134_4", family=binomial(link='logit'), data=mydata, nrep = 1000, seed=12345))

**3. Classification and regression tree (CART) analysis was conducted to screen out additionally important genetic variants for obesity risk. The analytical code for R v4.2.3 is as follows:**

library(rpart)

library(rpart.plot)

tree_model <- rpart(obesity ~ rs1349419+rs2167270+rs11208659+rs1137100+

rs1137101+rs6713532+rs16141+rs6127698+rs3746619+

rs17782313+rs12970134+rs8087522,

data = mydata, method = "class", parms = list(split="gini" ),

control = rpart.control(minsplit = 100, minbucket = 50, cp=0.001,

maxdepth = 5, xval = 10 ))

rpart.plot(tree_model)

**4. The risk for each subgroup in the CART was evaluated by comparison with the subgroup with the lowest proportion of cases, using logistic regression with the same adjustment. The analytical code for SAS 9.4 is as follows:**

**data** subgroup;

set mydata;

if missing(rs17782313) or missing(rs1349419) or missing(rs6713532) or missing(rs8087522) or missing(rs1137101) or missing(rs16141) then delete;

if rs17782313="TT" and rs1349419="GG" and rs8087522="GG" and rs1137101^="GG" then subgroup=**1**;

if rs17782313="TT" and rs1349419="GG" and rs8087522^="GG" then subgroup=**2**;

if rs17782313="TT" and rs1349419^="GG" then subgroup=**3**;

if rs17782313^="TT" and rs6713532="CC" and rs1137101="GG" then subgroup=**4**;

if rs17782313="TT" and rs1349419="GG" and rs8087522="GG" and rs1137101="GG" and rs16141^="GG" then subgroup=**5**;

if rs17782313="TT" and rs1349419="GG" and rs8087522="GG" and rs1137101="GG" and rs16141="GG" then subgroup=**6**;

if rs17782313^="TT" and rs6713532^="CC" then subgroup=**7**;

if rs17782313^="TT" and rs6713532="CC" and rs1137101^="GG" then subgroup=**8**;

**run**;

**proc** **logistic** data=subgroup descending ;

class subgroup sex maternal paternal incomes /param=ref Ref=first ;

model obesity=subgroup age sex maternal paternal incomes; **run**;

**5. Multivariate logistic regression model were applied to estimate the association between genetic risk score (GRS) and obesity risk. The analytical code for SAS 9.4 is as follows:**

**data** data_grs; set mydata;

if rs1137101="GG" then w101=**0**; else if rs1137101="AG" then w101=**1**; else if rs1137101="AA" then w101=**2**;

if rs6713532="CC" then w532=**0**; else if rs6713532="CT" then w532=**1**; else if rs6713532="TT" then w532=**2**;

if rs17782313="TT" then w313=**0**; else if rs17782313="CT" then w313=**1**; else if rs17782313="CC" then w313=**2**;

if rs12970134="GG" then w134=**0**; else if rs12970134="AG" then w134=**1**; else if rs12970134="AA" then w134=**2**;

grs=w313+w134+w101+w532;

if missing(w313) or missing(w134) or missing(w101) or missing(w532) then grs=**.**;

if grs=**0** then grs_cart=**1**;

if grs in(**1**,**2**) then grs_cart=**2**;

if grs in(**3**,**4**,**5**,**6**) then grs_cart=**3**;

if grs_cart in(**2**,**1**) then grs_cartcart=**1**;

if grs_cart in(**3**) then grs_cartcart=**2**;

wgrs=w532***0.044**+w313***0.268**+w134***0.217**+w101***0.110**;

if missing(w313) or missing(w134) or missing(w101) or missing(w532) then wgrs=**.**;

if wgrs=**.** then wgrs_cart=**.**;

else if wgrs<**0.044** then wgrs_cart=**1**; *Q1;

else if wgrs<**0.485** then wgrs_cart=**2**; *Q3;

else wgrs_cart=**3**;

if wgrs_cart in(**2**,**1**) then wgrs_cartcart=**1**;

if wgrs_cart in(**3**) then wgrs_cartcart=**2**;

if wgrs=**.** then wgrs_cart1=**.**;

else if wgrs=**0** then wgrs_cart1=**1**; else if wgrs<**0.1** then wgrs_cart1=**2**; else if wgrs<**0.2** then wgrs_cart1=**3**; else if wgrs<**0.3** then wgrs_cart1=**4**; else if wgrs<**0.4** then wgrs_cart1=**5**; else if wgrs<**0.5** then wgrs_cart1=**6**; else if wgrs<**0.6** then wgrs_cart1=**7**;

else if wgrs<**0.7** then wgrs_cart1=**8**; else if wgrs<**0.8** then wgrs_cart1=**9**; else if wgrs<**0.9** then wgrs_cart1=**10**; else if wgrs<**1.0** then wgrs_cart1=**11**; else wgrs_cart1=**12**;

**run**;

%let data=data_grs; %let x=grs_cart; %let y=obesity;

* *x* was then replaced by GRS_cartcart, wGRS_cart, and wGRS_cartcart, respectively;

**proc** **logistic** desc data=&data;

class &x(ref='1') ;

model &y(ref='0')=&x ;

title"Low as ref";

**run**;

**proc** **logistic** desc data=&data;

class sex maternal paternal incomes &x(ref='1') ;

model &y(ref='0')=&x sex maternal paternal incomes ;

title"Adjusted low as ref";

**run**;

%let data=data_grs; %let x=grs; %let y=obesity;

* *x* was then replaced by wGRS_cart1;

**proc** **logistic** desc data=&data;

model &y(ref='0')=&x ;

title"Per score increment";

**run**;

**proc** **logistic** desc data=&data;

model &y(ref='0')=&x age sex maternal paternal incomes ;

title"Adjusted per score increment";

**run**;

**6. Association between GRS (high vs. low/medium) and obesity risk stratified separately by sex and age group, with heterogeneity between regression coefficients tested by the *Z* test. The analytical code for SAS 9.4 is as follows:**

**data** boys; set data_grs; if sex=**0** and not missing(grs); **run**;

**data** girls; set data_grs; if sex=**1** and not missing(grs); **run**;

%let data=boys; %let x=grs_cartcart; %let y=obesity;

*data was sequentially replaced with 'girls', and *x* was then replaced by wGRS_cartcart, respectively;

**proc** **logistic** data=&data desc;

model &y(ref='0')=&x; **run**;

**proc** **logistic** data=&data desc;

class maternal paternal incomes /param=ref Ref=first ;

model &y(ref='0')=&x age maternal paternal incomes; **run**;

**data** younger;

set data_grs;

if **7**<=age<=**8** and not missing(grs); **run**;

**data** older;

set data_grs;

if **11**<=age<=**18** and not missing(grs); **run**;

%let data=younger; %let x=grs_cartcart; %let y=obesity;

*data was sequentially replaced with 'older', and *x* was then replaced by wGRS_cartcart, respectively;

**proc** **logistic** data=&data desc;

model &y(ref='0')=&x; **run**;

**proc** **logistic** data=&data desc;

class maternal paternal incomes sex /param=ref Ref=first ;

model &y(ref='0')=&x sex maternal paternal incomes;

**run**;

***Z-test was employed to assess the significance of stratified analyses;**

**data** z_test;

input b1 b2 se1 se2 n1 n2 Label $25. ;

bdiff=(b2-b1);

sediff=sqrt(se1****2**+se2****2**);

z=bdiff/sediff; z_jue=ABS(z);

P= exp(-**0.717***z_jue-**0.416***(z_jue****2**));

CI_Lower=bdiff-**1.96***sediff; CI_Upper=bdiff+**1.96***sediff;

CARDS;

0.3216 0.3858 0.1126 0.1639 1601 748 gender_unw

0.63 0.2793 0.2228 0.1024 442 1907 age_unw

0.2979 0.2483 0.1109 0.161 1601 748 gender_w

0.48 0.2334 0.2136 0.1011 442 1907 age_w;

**PROC** **print**; var b1 b2 bdiff sediff z p Label; id;

title 'Difference between two independent slopes';

**7. Crossover analysis was used to evaluate the joint effects of genetic risk scores and lifestyle behaviors on obesity risk. The analytical code for SAS 9.4 is as follows:**

%let x1=grs_cartcart; %let x2=Physical; %let y=obesity;

**x*1 was sequentially replaced with wgrs_cartcart, and *x*2 was then replaced by ‘Sleep’, respectively;

**data** Crossover;

set data_grs;

if &x1=**1** and &x2=**0** then group=**1**;

if &x1=**1** and &x2=**1** then group=**2**;

if &x1=**2** and &x2=**0** then group=**3**;

if &x1=**2** and &x2=**1** then group=**4**;

**run**;

**proc** **freq** data=Crossover;

table group*obesity / norow nocol nopercent;

**run**;

**proc** **logistic** data=Crossover descending ;

class group sex maternal paternal incomes /param=ref Ref=first ;

model obesity(ref='0')=group sex maternal paternal incomes ;

**run**;

**8. The relative excess risk of interaction (*RERI*) and the attributable proportion of interaction (*AP*) were calculated to characterize the potential interaction between lifestyle behaviors and GRSs on the additive scale. The analytical code for R v4.2.3 is as follows:**

1. **Unweighted GRS – sleep duration:**

## RERI ##

set.seed(12345)

reri <- function(datsam,indices){d<-datsam[indices,]

fitlr<-glm(formula = obesity ~ factor(grs_cartcart)*factor(sleep) + age + sex + maternal + paternal + incomes, data = d , family=binomial(link='logit'))

reri<-exp(fitlr$coef[10] + fitlr$coef[2] + fitlr$coef[3]) - exp(fitlr$coef[2]) - exp(fitlr$coef[3]) +1

reri}

outs<- boot(data=mydata , statistic=reri, R=1000)

print(outs)

boot.ci(outs,conf=0.95, type="perc")

## AP ##

set.seed(12345)

ap<-function(datsam,indices){d<-datsam[indices,]

fitlr<-glm(formula = obesity ~ factor(grs_cartcart)*factor(sleep) + age + sex + maternal + paternal + incomes, data = d , family=binomial(link='logit'))

ap<-(exp(fitlr$coef[10] + fitlr$coef[3] + fitlr$coef[2]) - exp(fitlr$coef[3]) -exp(fitlr$coef[2]) + 1) / exp(fitlr$coef[10] + fitlr$coef[3] + fitlr$coef[2])

ap}

outs<- boot(data=mydata, statistic=ap, R=1000)

print(outs)

boot.ci(outs,conf=0.95, type="perc")

## IOR ##

set.seed(12345)

ior<-function(datsam,indices){d<-datsam[indices,]

fitlr<- formula = obesity ~ factor(grs_cartcart)*factor(sleep) + age + sex + maternal + paternal + incomes, data = d , family=binomial(link='logit'))

ior<-exp(fitlr$coef[10] + fitlr$coef[3] + fitlr$coef[2]) / (exp(fitlr$coef[3]) * exp(fitlr$coef[2]))

ior}

outs<- boot(data=mydata, statistic=ior, R=1000)

print(outs)

boot.ci(outs,conf=0.95, type="perc")

1. **Unweighted GRS – physical activity:**

## RERI ##

set.seed(12345)

reri <- function(datsam,indices){d<-datsam[indices,]

fitlr<-glm(formula = obesity ~ factor(grs_cartcart)*factor(physical) + age + sex + maternal + paternal + incomes, data = d , family=binomial(link='logit'))

reri<-exp(fitlr$coef[10] + fitlr$coef[2] + fitlr$coef[3]) - exp(fitlr$coef[2]) - exp(fitlr$coef[3]) +1

reri}

outs<- boot(data=mydata , statistic=reri, R=1000)

print(outs)

boot.ci(outs,conf=0.95, type="perc")

## AP ##

set.seed(12345)

ap<-function(datsam,indices){d<-datsam[indices,]

fitlr<-glm(formula = obesity ~ factor(grs_cartcart)*factor(physical) + age + sex + maternal + paternal + incomes, data = d , family=binomial(link='logit'))

ap<-(exp(fitlr$coef[10] + fitlr$coef[3] + fitlr$coef[2]) - exp(fitlr$coef[3]) -exp(fitlr$coef[2]) + 1) / exp(fitlr$coef[10] + fitlr$coef[3] + fitlr$coef[2])

ap}

outs<- boot(data=mydata, statistic=ap, R=1000)

print(outs)

boot.ci(outs,conf=0.95, type="perc")

## IOR ##

set.seed(12345)

ior<-function(datsam,indices){d<-datsam[indices,]

fitlr<- formula = obesity ~ factor(grs_cartcart)*factor(physical) + age + sex + maternal + paternal + incomes, data = d , family=binomial(link='logit'))

ior<-exp(fitlr$coef[10] + fitlr$coef[3] + fitlr$coef[2]) / (exp(fitlr$coef[3]) * exp(fitlr$coef[2]))

ior}

outs<- boot(data=mydata, statistic=ior, R=1000)

print(outs)

boot.ci(outs,conf=0.95, type="perc")

1. **Weighted GRS – sleep duration:**

## RERI ##

set.seed(12345)

reri <- function(datsam,indices){d<-datsam[indices,]

fitlr<-glm(formula = obesity ~ factor(wgrs_cartcart)*factor(sleep) + age + sex + maternal + paternal + incomes, data = d , family=binomial(link='logit'))

reri<-exp(fitlr$coef[10] + fitlr$coef[2] + fitlr$coef[3]) - exp(fitlr$coef[2]) - exp(fitlr$coef[3]) +1

reri}

outs<- boot(data=mydata , statistic=reri, R=1000)

print(outs)

boot.ci(outs,conf=0.95, type="perc")

## AP ##

set.seed(12345)

ap<-function(datsam,indices){d<-datsam[indices,]

fitlr<-glm(formula = obesity ~ factor(wgrs_cartcart)*factor(sleep) + age + sex + maternal + paternal + incomes, data = d , family=binomial(link='logit'))

ap<-(exp(fitlr$coef[10] + fitlr$coef[3] + fitlr$coef[2]) - exp(fitlr$coef[3]) -exp(fitlr$coef[2]) + 1) / exp(fitlr$coef[10] + fitlr$coef[3] + fitlr$coef[2])

ap}

outs<- boot(data=mydata, statistic=ap, R=1000)

print(outs)

boot.ci(outs,conf=0.95, type="perc")

## IOR ##

set.seed(12345)

ior<-function(datsam,indices){d<-datsam[indices,]

fitlr<- formula = obesity ~ factor(wgrs_cartcart)*factor(sleep) + age + sex + maternal + paternal + incomes, data = d , family=binomial(link='logit'))

ior<-exp(fitlr$coef[10] + fitlr$coef[3] + fitlr$coef[2]) / (exp(fitlr$coef[3]) * exp(fitlr$coef[2]))

ior}

outs<- boot(data=mydata, statistic=ior, R=1000)

print(outs)

boot.ci(outs,conf=0.95, type="perc")

1. **Weighted GRS – physical activity:**

## RERI ##

set.seed(12345)

reri <- function(datsam,indices){d<-datsam[indices,]

fitlr<-glm(formula = obesity ~ factor(wgrs_cartcart)*factor(physical) + age + sex + maternal + paternal + incomes, data = d , family=binomial(link='logit'))

reri<-exp(fitlr$coef[10] + fitlr$coef[2] + fitlr$coef[3]) - exp(fitlr$coef[2]) - exp(fitlr$coef[3]) +1

reri}

outs<- boot(data=mydata , statistic=reri, R=1000)

print(outs)

boot.ci(outs,conf=0.95, type="perc")

## AP ##

set.seed(12345)

ap<-function(datsam,indices){d<-datsam[indices,]

fitlr<-glm(formula = obesity ~ factor(wgrs_cartcart)*factor(physical) + age + sex + maternal + paternal + incomes, data = d , family=binomial(link='logit'))

ap<-(exp(fitlr$coef[10] + fitlr$coef[3] + fitlr$coef[2]) - exp(fitlr$coef[3]) -exp(fitlr$coef[2]) + 1) / exp(fitlr$coef[10] + fitlr$coef[3] + fitlr$coef[2])

ap}

outs<- boot(data=mydata, statistic=ap, R=1000)

print(outs)

boot.ci(outs,conf=0.95, type="perc")

## IOR ##

set.seed(12345)

ior<-function(datsam,indices){d<-datsam[indices,]

fitlr<- formula = obesity ~ factor(wgrs_cartcart)*factor(physical) + age + sex + maternal + paternal + incomes, data = d , family=binomial(link='logit'))

ior<-exp(fitlr$coef[10] + fitlr$coef[3] + fitlr$coef[2]) / (exp(fitlr$coef[3]) * exp(fitlr$coef[2]))

ior}

outs<- boot(data=mydata, statistic=ior, R=1000)

print(outs)

boot.ci(outs,conf=0.95, type="perc")
